# Routine administration of neostigmine after recovery of spontaneous breathing versus neuromuscular monitor-guided administration of neostigmine in pediatric patients: a parallel, randomized, controlled study

**DOI:** 10.1186/s13063-023-07066-w

**Published:** 2023-01-07

**Authors:** Lei Yang, Na Hu, Hong Chang, Di Yang, Yunxia Zuo

**Affiliations:** 1 Department of Anesthesiology, West China Hospital, Sichuan University, Guoxuexiang 37, Chengdu, 610041 China; 2 Department of Anesthesiology, The Affiliated Hospital of Southwest Medical University, Luzhou, China; 3 Department of Anesthesiology, Sichuan Provincial Academy of Medical Sciences, Sichuan Provincial People’s Hospital, Chengdu, China

**Keywords:** Neostigmine, Anesthesia recovery period, Neuromuscular monitoring, Pediatric anesthesia, Randomized controlled trial

## Abstract

**Background:**

Neostigmine used to reverse the muscle relaxants should be guided by neuromuscular monitoring, as the degree of spontaneous pre-reversal recovery is the key to success to reverse the neuromuscular block. But neuromuscular monitoring is not always available for some patients during anesthesia and, in consequence, we need to use other clinical judgment to guide the use of neostigmine to reverse the neuromuscular block. In this trial, we aimed to evaluate the incidence of residual neuromuscular blockade (rNMB) in pediatric patients with routine use of neostigmine after recovery of spontaneous breathing compared with the patients with the use of neostigmine guided by neuromuscular monitoring.

**Methods:**

A parallel, randomized, controlled noninferiority study was conducted. We enrolled aged 3 months to 12 years old patients who underwent inguinal hernia repair under general anesthesia. The enrolled patients were randomly divided into experimental and control groups. After surgery, children in the experimental group were given 0.02 mg/kg neostigmine after recovery of spontaneous breathing. Children in the control group were given 0.02 mg/kg neostigmine when the train-of-four (TOF) ratio was between 0.4 and 0.9. However, no neostigmine was administered if the TOF ratio was higher than 0.9. The primary outcome was the incidence of rNMB after extubation (TOF ratio < 0.9). Secondary outcomes included the incidence of neostigmine-induced muscle paralysis, end of surgery – extubation interval, end of surgery – exit OR interval, the length of stay in the PACU, the incidence of hypoxia in the PACU, the number of children who required assisted ventilation during the PACU stay, and neostigmine-related adverse events.

**Results:**

A total of 120 children were included in this study, with 60 in the experimental group and 60 in the control group. There was no significant difference in the incidence of rNMB after extubation between the groups (45/60 vs 44/60, RR 1.02 [95% CI, 0.83 to 1.26], *p* = 0.84). There was no neostigmine-induced muscle paralysis in either group. Adverse events were similar occurred in both groups. However, time from end of the surgery to leaving the operating room was earlier in the experimental group than in the control group (13.6 ± 5.2 vs 15.7 ± 5.6 min, MD −2.10 min [95% CI, −3.70 to −0.50], *p* = 0.04). The risk ratio of the incidence of TOF ratio < 0.3 for the experimental group was 31.12 (95%CI, 1.89 to 512.61) compared with the control group (12/60 vs 0/60, *p* = 0.00) in exploratory analysis.

**Conclusions:**

Recovery of spontaneous breathing could be used as a substitute of neuromuscular monitoring to guide neostigmine use in pediatric patients following minor surgeries. However, care should be taken for the residual neuromuscular block.

**Trial registration:**

Chinese Clinical Trial Registry ChiCTR-IOR-17012890. Registered on 5 October 2017

## Background

Application of muscle relaxants can improve the surgery and anesthetic conditions of patients undergoing general anesthesia, and they are commonly used in the clinic [1]. However, residual neuromuscular blockade (rNMB) caused by application of muscle relaxants (train-of-four (TOF) <0.9) [2] may also lead to many serious postoperative complications, such as hypoxia and upper respiratory obstruction, which seriously threaten the postoperative safety of patients [3]. Although the recovery time of muscle relaxation in children is shorter than that in adult patients [4], the incidence of rNMB can still be as high as 28.1% [5].

Neostigmine, which is the most commonly used antagonist of muscle relaxants in the clinic, is often used at a dose of 0.02–0.07 mg/kg. Common adverse events include bradycardia, nausea, and vomiting [6, 7]. The use of neostigmine is also reported to be associated with postoperative respiratory complications [8]. Additionally, neostigmine may increase the risk of airway collapse and may lead to muscle paralysis under the full recovery of neuromuscular block [9]. Thus, the degree of spontaneous pre-reversal recovery is the key to success to reverse the neuromuscular block by neostigmine [10]. Neuromuscular monitoring, recommended by the Association of Anaesthetists of Great Britain and Ireland when using muscle relaxants [11], is the most ideal way to determine the degree of spontaneous recovery of neuromuscular blockade and the dose of reversal agents to be administered [12]. But as shown in the previous studies, even in developed countries, neuromuscular monitoring is not available for some patients during anesthesia because of clinical habits or lack of a monitoring device [13, 14]. When neuromuscular monitoring is not available, we need to use other clinical judgment to guide the use of neostigmine to reverse the neuromuscular block. A previous review showed that when spontaneous breathing recovers, the TOF ratio is approximately 0.3 and 0.02mg/kg neostigmine is sufficient to antagonize shallow degrees of rNMB [15].

Therefore, we hypothesized that recovery of spontaneous breathing could be used as a substitute of neuromuscular monitoring to guide the use of neostigmine to reverse the neuromuscular block. We aimed to evaluate the incidence of rNMB in pediatric patients with routine use of neostigmine after recovery of spontaneous breathing compared with the patients with the use of neostigmine guided by neuromuscular monitoring in this randomized, controlled noninferiority trial.

## Methods

This was a single-center, parallel, randomized, controlled trial, and was approved by the Ethics Committee of West China Hospital, Sichuan University. The study was registered in the Chinese Clinical Trial Registry (registration number: ChiCTR-IOR-17012890). The hospital that carried out this study is a research hospital in western China. This study included pediatric patients who underwent inguinal hernia repair under general anesthesia, used a muscle relaxant during surgery, had American Society of Anaesthesiologists Physical Status I–II, and were aged 3 months to 12 years. Pediatric patients with neuromuscular junction disease, cardiovascular disease, respiratory disease, or liver disease, as well as those who used drugs that may affect neuromuscular function in the past 3 months, were excluded. The parents or guardians of the involved children signed informed consent before surgery. The random number table was generated by a software program (EXCEL 2010, Microsoft, USA) and was sealed in an opaque envelope. The study was double blinded to the data collectors and the parents or guardians of the participants.

Guardians were allowed to accompany their children to the operating room (OR) until the children are sedated. Hence no pre-anesthesia medication was used. Anesthesia was induced by propofol, fentanyl, and cis-atracurium, and then endotracheal intubation was performed. Sevoflurane was used for maintenance of anesthesia. An electrocardiogram, pulse oxygen saturation, non-invasive blood pressure, and end-tidal carbon dioxide were routinely monitored during surgery. The ventilator settings were 8–10 ml/kg for tidal volume, 12–20 bpm for the respiratory rate, and the ratio of inspiration time to expiration time was 1:2. Neuromuscular function of the patient was monitored immediately after induction of anesthesia, and monitoring continued until the patient exited the OR. The enrolled pediatric patients were divided into the experimental group and the control group. For children in the experimental group, 0.02 mg/kg neostigmine and 0.01 mg/kg atropine were routinely administered after recovery of spontaneous breathing. Anesthesiologists were blinded to the TOF ratio of children in the experimental group. Children in the control group were administered neostigmine under the neuromuscular monitoring guided after surgery. When the TOF ratio was between 0.4 and 0.9, 0.02 mg/kg neostigmine and 0.01 mg/kg atropine were administered. If the TOF ratio was >0.9, neostigmine and atropine were not provided. The extubation indications and the criteria for exiting the OR were under the control of the anesthesiologist who performed the anesthesia. Tidal volume, respiratory rate, end-tidal carbon dioxide, status of consciousness, and TOF ratios were recorded before extubation. After extubation, children were transferred to the post-anesthesia care unit (PACU). Children were followed up until 1 day after the surgery. The follow-up nurses were not aware of the grouping of the participants.

The primary outcome was the incidence of rNMB immediately after extubation: rNMB defined as the TOF ratio was <0.9). The secondary outcomes were as follows: (1) the incidence of neostigmine-induced muscle paralysis — the decrease in the TOF ratio after administration of neostigmine. (2) end of surgery - extubation interval — the time from the end of surgery to endotracheal extubation; (3) end of surgery - exit OR interval — the time from the end of the surgery to when the patient was transferred out of the OR; (4) the length of PACU stay; (5) the incidence of hypoxia in the PACU (pulse oxygen saturation <90%); (6) the number of children who required assisted ventilation during the PACU stay — the patients need mask ventilation, larynx mask or endotracheal intubation; (7) neostigmine-related adverse events, such as nausea, vomiting, and bradycardia.

According to the previous studies, the incidence of rNMB was 15% in children with routinely using neostigmine to reverse atracurium [16] and we assumed that no rNMB occurred in children with neostigmine reversal under the neuromuscular monitor guided [17]. Based on the values, a sample size of 120 was needed to detect a non-inferiority difference of 0.01 between the two groups, which provided 80% power with a one-sided 2.5% level of significance, considering a 15% dropout rate. The data were analyzed by IBM SPSS 19.0 (IBM Company, Armonk, NY, USA). The chi-square test was used for categorical variables and the *t*-test was used for continuous variables. The statistical difference was set at *p* < 0.05.

## Results

**Fig. 1:**
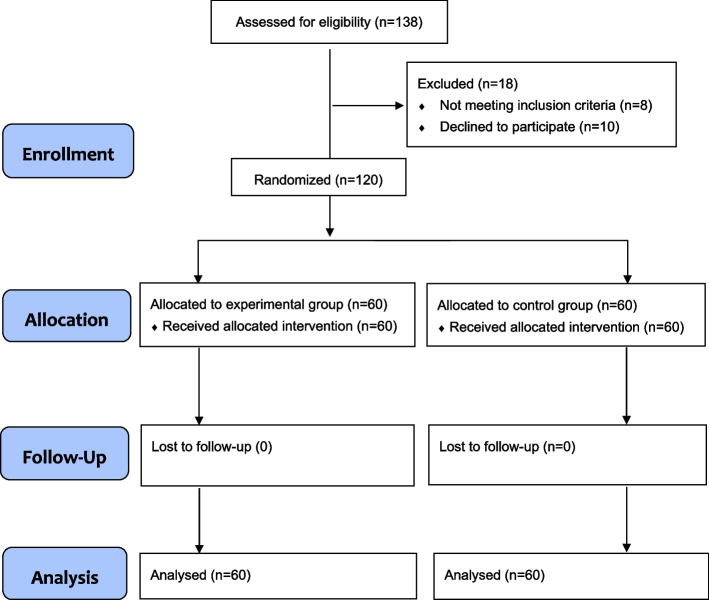
Study flowchart of the recruitment process

A total of 120 pediatric patients were included in the study (Fig. 1), with 60 cases in the experimental group and 60 cases in the control group. Data were collected from all included pediatric patients and were then analyzed. There were no significant differences in sex, age, body weight, surgical time, and anesthesia time between the two groups. There were also no differences in the dosages of muscle relaxants, opioids, antiemetic drugs, and respiration state before extubation between the two groups (Table 1). All the patients in the experimental group received neostigmine and 95% patients in the control group (57/60) received neostigmine.

**Table 1:** General information of included pediatric patients

Variables	Experimental group (***n***=60)	Control group (***n***=60)	***p-***value
**General characteristics**			
Sex (male/female), No.	48/12	45/15	0.51
Age (years), mean ± SD	3.5 ± 1.9	4.0 ± 2.2	0.29
Body weight (kg), mean ± SD	16.1 ± 4.3	17.1 ± 5.8	0.28
Surgical time (min), mean ± SD	18.1 ± 8.0	16.9 ± 6.2	0.38
Anesthesia time (min), mean ± SD	36.9 ± 9.9	35.2 ± 9.0	0.33
**Intraoperative Medications**			
Cis-atracurium (mg), mean ± SD	1.4 ± 0.5	1.6 ± 1.0	0.14
Fentanyl (ug), mean ± SD	38.9 ± 12.9	40.5 ± 11.6	0.48
Ondansetron (mg), mean ± SD	1.6 ± 0.7	1.8 ± 1.1	0.29
**Respiration state before extubation**			
Respiratory rate (bpm), mean ± SD	19.3 ± 5.6	19.4 ± 4.5	0.94
Tidal volume (ml), mean ± SD	124.9 ± 51.8	135.2 ± 60.3	0.32
Pulse oxygen saturation (%), mean ± SD	99.7 ± 0.9	99.9 ± 0.3	0.13
End-tidal carbon dioxide (mmHg), mean ± SD	45.6 ± 6.6	45.5 ± 6.0	0.94

*min* minute, *bpm* breath per minute

For the primary outcome, there was no significant difference in the incidence of rNMB after extubation between the groups (45/60 vs 44/60, RR 1.02 [95% CI, 0.83 to 1.26], *p* = 0.84). We performed a further exploratory analysis to compare the incidence of TOF ratio < 0.3 between groups. The risk ratio of the incidence of TOF ratio < 0.3 for the experimental group was 31.12 (95%CI, 1.89 to 512.61) compared with the control group (12/60 vs 0/60, *p* = 0.00) (Table 2).

**Table 2:** Primary and secondary outcomes

Outcomes	Experimental group (***n***=60)	Control group (***n***=60)	Effect estimated (95% CI)	***p***-value
**Primary outcomes**				
Cases of rNMB after extubation (TOF ratio <0.9), *n* (%)	45 (75.0)	44 (73.3)	1.02 (0.83 to 1.26)^a^	0.84
Cases of patients with TOF ratio < 0.3, *n* (%)	12 (20)	0 (0)	31.12 (1.89 to 512.61)^a^	0.00
**Secondary outcomes**				
Cases of neostigmine-induced muscle paralysis, *n* (%)	0 (0)	0 (0)	-	-
End of surgery – extubation interval (min), mean±SD	8.9 ± 5.1	9.6 ± 4.5	−0.70 (−2.42 to 1.02)^b^	0.40
End of surgery – exit OR interval (min), mean±SD	13.6 ± 5.2	15.7 ± 5.6	−2.10 (−3.70 to −0.50)^b^	0.04
The length of stay in the PACU (min), mean±SD	45.1 ± 15.6	46.0 ± 13.5	−0.90 (−6.12 to 4.32)^b^	0.73
Cases of hypoxia, *n* (%)				
In OR	2 (3.3)	1(1.7)	2.00 (0.19 to 21.47)^a^	0.56
In PACU	0 (0)	0 (0)	-	-
Cases of children who required assisted ventilation, *n* (%)				
In OR	2 (3.3)	1 (1.7)	2.00 (0.19 to 21.47)^a^	0.56
In PACU	0 (0)	0 (0)	-	-
Cases of bradycardia, *n* (%)				
In OR	0 (0)	0 (0)	-	-
In PACU	0 (0)	0 (0)	-	-
Cases of nausea and vomiting, *n* (%)				
In PACU	0 (0)	0 (0)	-	-
On the first postoperative day	1 (1.7)	0 (0)	3.00 (0.12 to 72.20)^a^	0.32

*rNMB* residual neuromuscular blockade, *min* minute, *OR* operating room, *PACU* postanesthesia care unit

^a^Effect estimated is the risk ratio (RR)

^b^Effect estimated is the mean difference (MD)

For the secondary outcomes, there was no neostigmine-induced muscle paralysis in either group. The end of surgery - exit OR interval in the experimental group was 2.1 min (95%CI, −3.70 to −0.50) shorter than that in the control group (13.6 ± 5.2 vs 15.7 ± 5.6 min, *p* = 0.04). The end of surgery – extubation interval in the experimental group was similar to the control group (8.9 ± 5.1 vs 9.6 ± 4.5 min, *p* = 0.40) and the length of stay in the PACU in the experimental group was also similar to the control group (45.1 ± 15.6 vs 46.0 ± 13.5, *p* = 0.73). After extubation, two cases in the experimental group and one case in the control group developed mild airway obstruction and hypoxia in the operating room. They all recovered after airway management of jaw thrust and improved after inhaling pure oxygen. During the PACU stay, no cases of hypoxia or airway obstruction occurred in either group. However, after surgery, nausea and vomiting occurred in one case in the experimental group on the first postoperative day (Table 2).

## Discussion

The incidence of rNMB after extubation in pediatric patients with routine use of neostigmine after recovery of spontaneous breathing (experimental group) was not significantly different compared with that in pediatric patients who were provided neostigmine guided by the monitoring of neuromuscular function (control group). Although anesthesiologists were aware of the TOF ratios in the control group, rNMB still occur in 73.3% patients after extubation. A similar result was found in a previous cohort study, in which 64.7% patients had rNMB at tracheal extubation, despite neostigmine administration and qualitative neuromuscular function monitor used [18]. This may be due to the fact that anesthesiologists recognized rNMB according to clinical signs of adequate ventilation such as tidal volume, respiratory rate, oxygen saturation, and end-expiratory carbon dioxide, rather than the neuromuscular monitoring. However, neuromuscular monitoring becomes important when ventilation is inadequate because anesthesiologists distinguish whether this is caused from rNMB. Although the incidence of rNMB was similar in both groups, the incidence of severe rNMB (TOF < 0.3) after extubation was much higher in the experimental group. It indicated the value of neuromuscular monitoring to prevent tracheal extubation under severe rNMB. In addition, considering the process of recovery from muscle paralysis, the shorter end of surgery – exit OR interval indicated shorter observation in OR in the experimental group. It hinted that there were potential risks for transferring the patients with severe rNMB in the experimental group, although the adverse events in the PACU were similar in both groups. These findings suggest the clinical importance of neuromuscular monitoring in identifying and preventing rNMB, which is worthy of further study.

The use of muscle relaxant antagonists at the end of the surgery was reported to be associated with a decreased risk of mortality [19], but when considering the induced muscle paralysis of neostigmine, ineffectiveness under the deep block and related adverse events, such routinely use at the end of the surgery remains controversial [12, 20]. There was no neostigmine-induced muscle paralysis in either of the groups in our study. A similar result was found in a previous study that administration of neostigmine during the reversal of a neuromuscular blockade did not lead to muscle paralysis in adult patients [21]. Besides, our study showed that the incidence of adverse events including postoperative nausea and vomiting was relatively low in both groups, and there was no significant difference between the two groups. There are many factors that affect nausea and vomiting, including age, disease history, anesthetic drugs, and the type of surgery. All the pediatric patients who were included in this study underwent hernia repair. The risk of postoperative nausea and vomiting in hernia repair is not high. Furthermore, anti-nausea and vomiting drugs were routinely used to prevent postoperative nausea and vomiting. Therefore, the risks of related postoperative adverse events in this study were low.

This study has some limitations. First, awake pediatric patients are not tolerant of neuromuscular monitoring. Therefore, monitoring of neuromuscular function was not carried out during the PACU stay. Consequently, the incidence of rNMB in the two groups during the PACU stay was unknown. Second, all the pediatric patients in this study were transferred to the PACU after surgery for observation. Therefore, the results of this study do not apply to pediatric patients who need to return to the ward directly after surgery.

## Conclusions

Recovery of spontaneous breathing could be used as a substitute of neuromuscular monitoring to guide neostigmine use in pediatric patients following minor surgeries. However, care should be taken for the residual neuromuscular block.

## Data Availability

The datasets used and/or analyzed during the current study are available from the corresponding author on reasonable request.

## Abbreviations

rNMB: Residual neuromuscular blockade
TOF: Train-of-four
OR: Operating room
PACU: Post-anesthesia care unit
